# New Fmoc-Amino Acids/Peptides-Based Supramolecular Gels Obtained through Co-Assembly Process: Preparation and Characterization

**DOI:** 10.3390/polym14163354

**Published:** 2022-08-17

**Authors:** Alexandra Croitoriu, Loredana Elena Nita, Alina Gabriela Rusu, Alina Ghilan, Maria Bercea, Aurica P. Chiriac

**Affiliations:** “Petru Poni” Institute of Macromolecular Chemistry, 41-A Grigore Ghica Voda Alley, 700487 Iasi, Romania

**Keywords:** bionic hydrogel, Fmoc peptide, gelator, supramolecules

## Abstract

One of the methods of obtaining supramolecular gels consists of the possibility of self-assembly of low molecular weight gelators (LMWGs). However, LMWG-based gels are often difficult to handle, easy to destroy and have poor rheological performance. In order to improve the gels’ properties, the LMWGs molecules are co-assembled, which induces more cross-links with more stable structures. Starting from these aspects, the present study refers to the preparation of a bionic hydrogel stabilized with a physiologically occurring, bifunctional biomolecule, L-lysine, co-assembled with other amino acids or peptides (such as a modified amino acid (Fmoc-serine or Fmoc-glutamic acid) or a tripeptide (Fmoc-Gly-Gly-Gly)) with the potential to support the repair of injuries or the age-related impaired structures or functions of living tissues. The introduction of a copartner aims to improve hydrogel characteristics from a morphological, rheological and structural point of view. On the other hand, the process will allow the understanding of the phenomenon of specific self-association and molecular recognition. Various characterization techniques were used to assess the ability to co-assemble: DLS, FT-IR, SEM and fluorescence microscopy, rheology and thermal analysis. Studies have confirmed that the supramolecular structure occurs through the formation of inter- and intramolecular physical bonds that ensure the formation of fibrils organized into 3D networks. The rheological data, namely the G′ > G″ and tan δ approximately 0.1–0.2 gel-like behavior observed for all studied samples, demonstrate and sustain the appearance of the co-assembly processes and the ability of the samples to act as LMWG. From the studied systems, the Fmoc–Lys–Fmoc_ Fmoc–Glu sample presented the best rheological characteristics that are consistent with the observations that resulted from the dichroism, fluorescence and SEM investigations.

## 1. Introduction

Supramolecular amino acid-based hydrogels are a class of soft materials with outstanding properties that faithfully mimic living tissues and provide a precise environment for the growth and development of cell cultures or the delivery of bioactive principles. Supramolecular gels formed by the self-assembly of low molecular weight gelators (LMWGs) find their potential applications in various biomedical fields, such as soft tissue engineering, drug administration, cell encapsulation, cell growth and so on [1]. In the biomedical field, LMWGs applications from the class of peptides and functionalized amino acids are mainly used. Peptides and amino acids possess intrinsic anti-inflammatory properties, due to their hydrophobicity, polarity and amphipathic behavior [2]. L-lysine is an amino acid with positively charged cationic groups which are responsible for the disruption of the bacterial membranes [3]. In addition, the antibacterial activity of Fmoc-Lys-Fmoc against gram-positive and gram-negative bacteria offers a wide variety of possible uses associated with biocompatibility. Fluorenylmethyloxycarbonyl (Fmoc)-protected amino acids or peptides have emerged as excellent building blocks for the development of self-assembled functional structures. Unlike most chemically formed gels, which form irreversible networks, physical gels form reversible networks that can participate in subsequent reactions of functionalization, having as well interesting self-healing properties [4]. In the last years, supramolecular hydrogels formed from LMWGs have attracted interest not only because of their applicability as new soft materials, but also due to their interesting properties generated through molecular organization that leads to highly ordered structures. LMWG compounds can induce hydrogelation, organogelation or both processes (ambidextrous), depending on the desired nature of the gel and the choice of the organic solvents [5]. The Fmoc-Lys-Fmoc includes an additional Fmoc group, protecting the L-lysine (Lys) side chain. Thus, additional bridges can be formed in the co-assembly processes by additional H-binding from the carbonyl group, aromatic and hydrophobic interactions from the Fmoc ring, and steric optimization from the methoxycarbonyl [5].

Different studies have focused on designing supramolecular systems containing two molecules capable of noncovalently interacting to form a fibrous network. Assuming that the interactions between different components are generated by complementary chemical groups, a range of possible assemblies can arise. The pioneering studies sustain that, depending on the preparation method or hydrophobicity, molecules of LMWGs self-assemble into one-dimensional fibers by self-sorting or co-assembling. According to studies realized by Adams et al. [6,7], the process of self-sorting can be reached using a pH-triggered approach, while LMWG has been assembled in the presence of a surfactant. For example, the Ulijn group [8] has demonstrated that mixing a dipeptide-based LWMG with an amino acid-based surfactant can lead to an orthogonal, disruptive, or cooperative assembly of the two components, depending on the choice of gelator and surfactant [7]. Likewise, Gazit et al. [5] sustain that the presence of the Fmoc group maintains the balance between hydrophobic and hydrophilic functionalities necessary for self-assembly.

Although hydrogels have already been obtained from various Fmoc mono-functionalized amino acids (especially phenylalanine (Phe) or Phe-Phe peptides) [9,10,11], the use of Fmoc-di-functionalized amino acids is recent. In this context, the importance of the second Fmoc fragment in double-functionalized Lys (Fmoc-Lys-Fmoc) has been demonstrated in its use as an LMWG. Unlike other functionalized amino acids, Fmoc-Lys-Fmoc has pH-controlled ambidextrous gelling [12]. It should be noted that Fmoc-Lys-Fmoc not only has two fluorenyl groups (which improved the co-assembling), but also has an additional –CO-NH group, which can participate in the formation of additional hydrogen bonds, also allowing the formation of cross-linking bridges with another amino acid. In addition, Lys has an inherently small alkyl chain [13], which is hydrophobic and is usually required in organogels formation [14,15,16,17].

The chemical functionality of amino acids plays a key role in the co-assembly process, offering the possibility of obtaining new materials with ordered structures and a promise for three-dimensional (3D) biological studies. The essential parameters of hydrogels with applicability in the biomedical field, in addition to biocompatibility, are the mechanical properties, the rate of degradation, the sensitivity to various external stimuli and also the porosity and the degree of swelling of the material [18]. The predictable change of these parameters depends directly on the partners entering the co-assembly process, as well as on the relationship between them. Bionic design consists of integrating information from biological systems into the design and development of new products in order to develop novel medical tools with good safety and efficacy profiles [19].

L-lysine is an amino acid physiologically present in the body and is recognized for playing a significant role in cell adhesion and collagen cross-linking [20]. It is of interest to obtain hydrogels by co-assembling two amino acids or an amino acid and a peptide, one of which is Fmoc-Lys-Fmoc, known for its ability to form (hydro)gels with antibacterial properties [21]. From a morphological, rheological and structural point of view, the introduction of a copartner aims to improve hydrogel properties resulting from the interaction between the two different components, and the process allows the understanding of the phenomenon specific to self-association and molecular recognition development, and also, to identify the driving forces involved in generating three-dimensional supramolecular structures of amino acids [22]. Considering these aspects, we designed a bionic hydrogel stabilized with a physiologically occurring bifunctional biomolecule, L-lysine, which has the potential to support the repair of injuries or the age-related impaired structures or functions of living tissues, together with a partner, such as a modified amino acid (Fmoc-serine or Fmoc-glutamic acid) or a tripeptide (Fmoc-Gly-Gly-Gly).

## 2. Materials and Methods

### 2.1. Materials

Fmoc–Lys–Fmoc–OH (C_36_H_34_N_2_O_6_, Mw = 590.66 g/mol, from Sigma-Aldrich (Darmstadt, Germany)), Fmoc-Ser-OH (C_18_H_17_NO_5_, Mw = 327.34 g/mol, from Alfa Aesar (Kandle, Germany)), Fmoc-Glu (C_20_H_19_NO_6_, Mw = 369.37 g/mol, from Alfa Aesar (Kandle, Germany)) amino acids and Fmoc-Gly-Gly-Gly-OH (C_21_H_21_N_3_O_6_, Mw = 411.41 g/mol, from Bachem (Bubendorf, Switzerland)) tripeptide were used as received. Sodium phosphate buffer (PBS, pH 7.4, 0.01 M) was prepared using monosodium phosphate (NaH_2_PO_4_ × 2H_2_O) and disodium phosphate (Na_2_H_2_PO_4_ × 7H_2_O) in distilled water via standard protocol. Dimethyl sulfoxide (DMSO) was acquired from Fluka (Buchs, Switzerland) and used as the solvent.

### 2.2. Methods

The preparation of hydrogels from Fmoc-Lys-Fmoc and X, the copartners, namely Fmoc-Gly-Gly-Gly and Fmoc-Ser or Fmoc-Glu, was performed by individually preparing two stock solutions and mixing them in different gravimetric ratios to obtain three co-assembled gel variants as presented in Table 1.

**Scheme 1:**
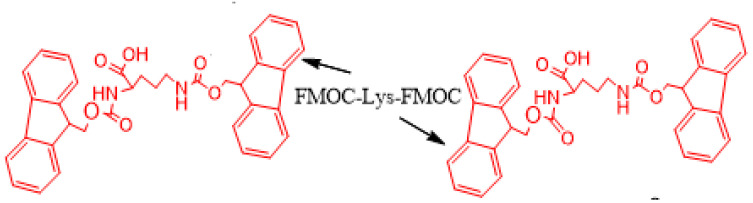
Idealized self-assembly process for Lys (in the case of pairs). One molecule of Lys is replaced with an X (copartner).

For the preparation of the solutions, the approach technique was the use of a polar solvent. Each copartner sample dissolved in DMSO was mixed until the solution became transparent, and a further 0.01 M phosphate buffer (pH = 7.4) was gradually added to the clear solution to avoid film formation on contact between them. Then, the Fmoc-Lys-Fmoc solution was added to the copartner solution in various ratios (Table 1), and the mixture was slightly homogenized (with a spatula) and left at room temperature for 24 h, after which the hydrogel formation was macroscopically verified by the inverted test tube (Table 2). The cross-linking was realized for co-assembled pairs, as is presented schematically in Scheme 1.

### 2.3. Characterization of the Prepared Gels

The synthesized gel samples were lyophilized prior to scanning electron microscopy (SEM), Fourier transform infrared (FTIR) spectroscopy, or thermal assay (TG/DTG), or stored in a solution at a concentration under gelling conditions for dynamic light scattering (DLS), ultraviolet-visible (UV-VIS), fluorescence and circular dichroism (CD) analyses.

#### 2.3.1. Characterization in Solution of the Prepared Gels

##### The Size Distribution Profile of Samples

DLS measurements were performed on a Malvern Zetasizer Nano equipped with a He-Ne laser (633 nm). Aqueous solutions were filtered using a Millipore membrane filter (0.45 µm pore size) before the measurements. The particle size distribution was derived from a deconvolution of the measured intensity autocorrelation function by the “general purpose mode” (nonnegative linear least-squares) algorithm included in the software of the instrument.

##### Fluorescence Studies

Fluorescence measurements were recorded at room temperature with a Perkin Elmer fluorescence spectrophotometer. The excitation wavelength was 285 nm, and the emission and excitation slit widths were set at 6 and 3 nm, respectively. The fluorescence spectra were obtained in the range of 290–550 nm at an integration time of 1.0 s/nm. The measurements were performed by adding small aliquots of amino acid derivatives with a 0.0025% concentration.

##### CD Spectra

CD spectra of Fmoc-X derivatives, performed in an aqueous solution of 1.0 mg/mL, was registered with a Chirascan plus (Applied Photophysics Ltd, Leatherhead, United Kingdom) spectrometer by using 2 mm path lamellar cells. Thermal controller unit using a 0.1 mm quartz cell was set to 22 °C. The spectra of the samples were recorded from 400 to 230 nm. Each spectrum was obtained by averaging three scans and correcting for the blank.

##### UV-VIS Spectroscopy

Optical properties of the prepared solutions were obtained through UV-VIS reflectance spectra measured with the Analytik Jena SPECORD UV/Vis 210Plus spectrophotometer(Analytik Jena, Jena, Germany).

#### 2.3.2. Characterization of Gels in the Wet State

##### Rheological Studies

Rheological investigations were carried out by using an MCR 302 Anton-Paar rheometer with a plane-plane geometry (the upper plate diameter of 50 mm and a gap of 0.5 mm) and a Peltier device for temperature control. The wet hydrogel samples were investigated in dynamic rheological tests. Preliminary strain sweep tests (0.01–100%) were carried out in order to identify the linear viscoelastic regime. The storage (or elastic) modulus, G’, the loss (or viscous) modulus, G” and the complex viscosity, η* were determined in oscillatory frequency sweep tests from 0.1 rad/s to 100 rad/s at 25 °C and 37 °C. The loss tangent, tan δ, defined as G”/G’, ratio was monitored to identify the degree of viscoelasticity for each investigated sample.

#### 2.3.3. Characterization of Lyophilized Gels

##### FTIR Spectroscopy

FTIR spectra of the lyophilized gels were recorded from KBr pellets with a Vertex 70 spectrophotometer (Bruker, Ettlingen, Germany). The vibrational transition frequencies were reported in wavenumbers (cm^−1^) and the FTIR spectra were recorded at a 400–4000 cm^−1^ interval, at 4 cm^−1^ resolution.

##### TG/DTG Analysis

The thermal stability of the prepared lyophilized gels was evaluated by thermogravimetric analysis on a STA 449 F1 Jupiter apparatus (Netzsch, Selb, Germany). The samples, with weights ranging from 8 to 10 mg, were placed in an open Al_2_O_3_ crucible and thermally degraded. The temperature range was 30–670 °C in an N_2_ (99.99% purity) atmosphere. Runs were performed in a dynamic mode, with a heating rate of 10 °C/min under a gas flow of 40 mL/min. The thermogravimetric balance was calibrated on temperature and sensitivity with standard metals (In, Sn, Bi, Zn and Al) from 25 to 700 °C. Data collection was performed with Proteus^®^ software.

##### Morphology Analysis

Preparation of the samples for the morphological characterization by SEM investigation was created by freezing the preformed gels with liquid nitrogen and then storing the freeze-dried gels for 48 h until all the water was sublimated. SEM investigations were performed on samples fixed in advance by means of colloidal copper supports. The samples were spray-coated with a thin layer of gold (K Emitech550X). The area covered was examined by using a scanning electron microscope, type Quanta 200, which operates at 30 kV with secondary electrons in high vacuum mode.

## 3. Results

The realization of gel-type systems through intra- and intermolecular physical connections ensures through the functional groups the formation of self-assembly processes of inclusive network structures with self-healing capacity, and the absence of chemical cross-linkers guarantees the biocompatible nature of hydrogel variants based on LMWGs. At the same time, such a physical connection does not provide the desired resistance in the network. As a result, the identification of pairs of compounds that increase the chances of physical cross-linking and lead to improved gel properties can only be beneficial. The following confirms the achievement of this objective through the studied components.

### 3.1. Hydrogelation Systems

The hydrogelation of Fmoc-X systems performed in DMSO was confirmed by the conventional vial inversion method as it is illustrated in Table 2. As it is well known, Fmoc-Lys-Fmoc forms stable gels [12], and we used different co-assembly variants in order to conclude on each tested alternative of gelation. According to Table 1, for each system, three gravimetric ratios between Fmoc-Lys-Fmoc and the second compound were chosen. Samples with a higher amount of Fmoc-Lys-Fmoc (15_1 compared to the second component) show better viscoelastic behavior and a translucent aspect, while samples with low Fmoc-Lys-Fmoc content are less stable and opaquer.

### 3.2. Co-Assembly Processes

#### 3.2.1. FTIR Spectra

**Figure 1:**
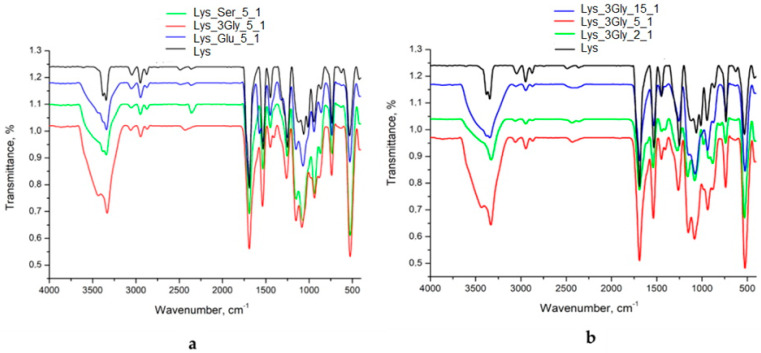
FTIR spectra of (**a**) the amino acid/peptide systems that formed supramolecular gels (at a constant ratio of 5_1) and (**b**) the pairs, Lys_3Gly, at different ratios between components.

Figure 1a shows the FTIR spectra of the freeze-dried gels obtained by the co-assembly of Fmoc-Lys-Fmoc with other amino acids or tripeptides (constant ratio of 5_1) compared with Fmoc-Lys-Fmoc. Figure 1b shows the FTIR spectra of the pairs, Lys_3Gly, at three different ratios between components.

The wide bands from 3400 cm^−1^ are characteristics of the OH groups present in the structure. The presence of C-H groups is highlighted by the corresponding peak at 3046 cm^−1^ in the Lys spectrum. This peak is shifted to approximately 3055 cm^−1^ for freeze-dried co-assembled gels, which confirms the interaction between components. The specific vibrations of C=O from the carboxyl group present at 1700–1690 cm^−1^ provide information on the formation of hydrogen bonds. The presence of amide I (νC = O) bands is observed at 1689 cm^−1^ and amide II (δN-H and νC-N) at 1530 cm^−1^. These regions which are assigned to amide I and amide II are often used for analyzing secondary structures [23,24]. The effect of the ratio between the components (Figure 1b) is confirmed by the shift of the absorption bands from 1695 cm^−1^ (for Lys_3Gly_15_1) to 1705 cm^−1^ (for Lys_3Gly_2_1) that points out the involvement of a larger number of carboxyl groups in the formation of intra- and intermolecular hydrogen bonds. The peak from 1530 to 1535 cm^−1^ is specific to the amino bond of the amide group and appears in all the analyzed samples. The presence of bands in the 1200–1300 cm^−1^ domains corresponds to the C-O and C-N stretching vibrations. Another important region in the FTIR spectra of the co-assembled freeze-dried gels is the amide III band recorded at 1250–1260 cm^−1^. The intensity of this band (νC-N and δN-H) is reduced in the case of co-assembled structures, while the intensity of the band in the FTIR spectrum of the control compound (Fmoc-Lys-Fmoc) is obviously higher. The decrease in their intensity as the amount of Fmoc-Gly-Gly-Gly increases is attributed to a possible molecular reorientation of the peptide. The absorption bands in the region 950–730 cm^−1^ are specific to the groups of aromatic rings in the FMOC structure [25].

#### 3.2.2. Thermal Analysis

Table 3 presents the main parameters of the thermal decomposition of the synthesized hydrogels for a 5_1 ratio between Fmoc-Lys-Fmoc and its copartners. Fmoc-amino acids self-assemble through a combination of weak hydrophobic forces, leading to π-π stacking of the fluorenyl groups further stabilized by hydrogen bonding interactions [26]. The amino acid structure indicates the possibility of decomposition in CO_2_, CO, HCN and NH_3_, as the major products and H2O as a minor product in the 27–700 °C temperature range. Furthermore, in the case of the co-assembled gels, the weight loss will be influenced by the side-chain groups of Gly, Ser and Glu [27].

**Figure 2:**
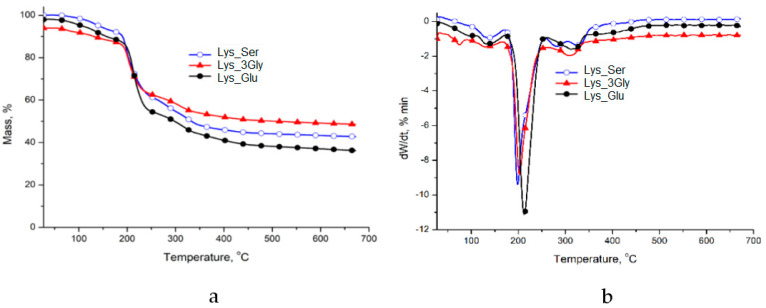
TG (**a**) and DTG (**b**) of the amino acid/peptide systems that formed supramolecular gels at a constant ratio (Fmoc-Lys-Fmoc: X = 5_1).

The thermal behavior of the freeze-dried gels is depicted in Figure 2. According to Figure 2 and Table 3, four degradation stages occur for all analyzed gels. The first stage of weight loss is associated with water molecules or solvent residues’ evaporation from gel structures at temperatures below 150 °C.

From about 200 °C, a significant weight loss occurs in the third stage of gels decomposition (21.03 wt.% for Lys _Ser, 26.18 wt.% for Lys_3Gly and 34.55 wt.% for Lys_Glu). This process arises due to the cleavage and decomposition of hydroxyl, amine and carboxyl groups. Likewise, the separation of the π-π stacking planes could influence the thermal stability of gels. Due to the relatively similar supramolecular interactions found in all studied hydrogels, the TGA thermal curves also show a similar behavior [28]. Nevertheless, the residue percent corroborated with T_10_ and T_20_ parameters indicates a better thermostability for Lys_3Gly (54.58 wt%) gel compared to Lys _Ser (42.77 wt.%) and Lys_Glu (38.17 wt.%) samples.

#### 3.2.3. DLS Studies

**Figure 3:**
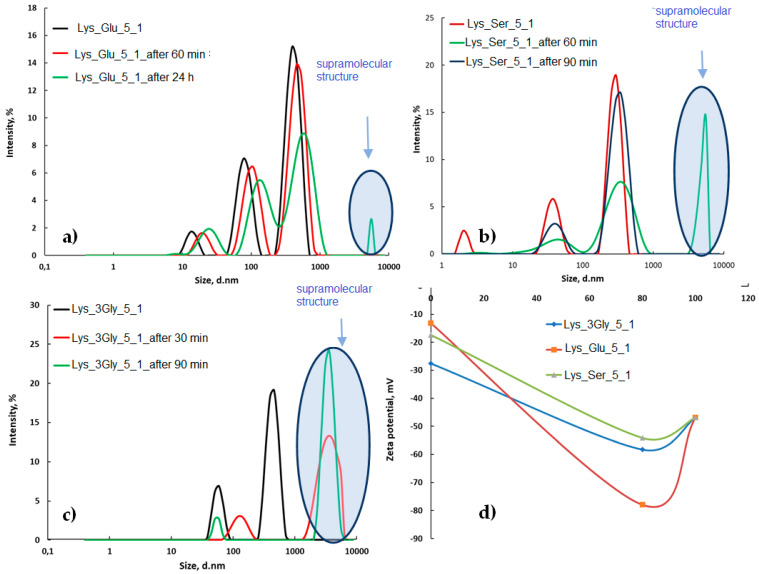
Size variation with time for (**a**) Lys_Glu_5_1, (**b**) Lys_Ser_5_1, (**c**) Lys_3Gly_5_1, and (**d**) zeta-potential variation of the co-assembled structures compared to the starting compounds.

The DLS analyses illustrated in Figure 3 were performed on LMWG solutions before the formation of supramolecular gels.

DLS studies confirm that the supramolecular structure [29] occurs over time, this aspect is highlighted by the appearance of a characteristic peak of agglomerations at values of over 5000 nm. The behavior is different depending on the amino acid or peptide used together with Lys in the co-assembly process. Thus, when the pair is Glu (Figure 3a), the reaction is slower, and although associations are observed in the first 60 min, they are more obvious after 24 h. In the case of Ser and 3Gly peptides (Figure 3b,c), the points of assembly appear from the first 30 min, being more obvious in the case of peptides (Figure 3c) due to the possibility of forming supplementary intra- and inter-linkages in this case. The zeta-potential (Figure 3d) of Lys is approximately −45 mV more negative than the zeta-potential of copairs, which varies between −12 mv for Glu to −28 mV for 3Gly. In the case of the co-assembled supramolecular structures, the zeta-potential increases considerably in the module and varies from −54 mV for Lys_Ser pairs to −78 mV for Lys-3Gly pairs. This higher value attests once again to the interactions that appear between the components and the formation of the supramolecular structure.

#### 3.2.4. Molecular Arrangement

**Figure 4:**
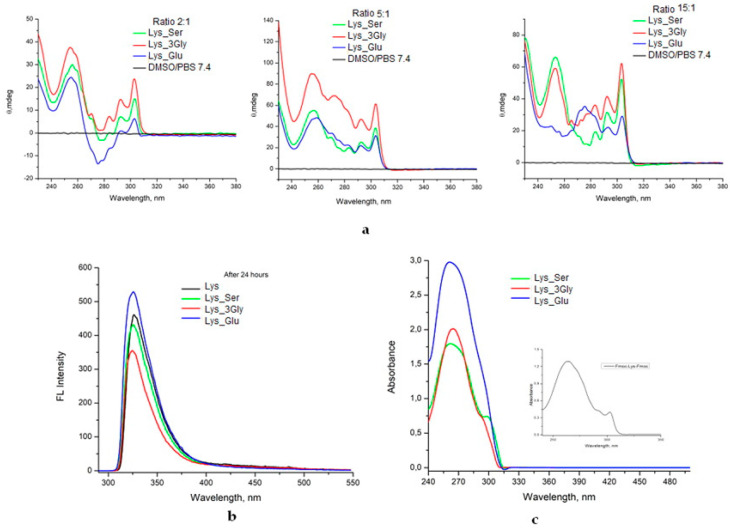
(**a**) CD spectra, (**b**) fluorescence spectra and (**c**) UV-VIS spectra for the co-assembled structures in solution.

The secondary structure of co-assembling in solution was investigated by circular dichroism (CD), fluorescence and UV-VIS spectroscopy (Figure 4).

Depending on the wavelength of the absorption of right or left circularly polarized light by optically active molecules, the CD technique highlights the conformation of uncoordinated structures (random coils) and ordered structures (α-helices and β-sheets). The main protein chromophores are peptide bonds, aromatic amino acids and disulfide bonds. The α carbon atom in the amino acid structure is the chiral center that influences optical activity. According to the previous studies [29], the peptide bond shows optical activity in the 250 nm region of the CD spectrum, while the peaks in the 260 nm region at 300 nm are attributed to aromatic groups.

The CD spectra of the solutions were recorded between 320 and 220 nm, and the corresponding dichroic signals are reported in Figure 4a. It can be seen that, depending on the ratio between the two co-assembled amino acids, different molecular arrangements are obtained. Peaks at 250 to 310 nm are associated with the π-π * transition of fluorenyl absorption [30]. In particular, the signal at 305 nm is an indicator of the coupling of fluorenyl chromophores in the formation of molecular assemblies as precursors of hydrogels. The peak at approximately 305 nm is more pronounced for assemblies where the amount of lysine is higher (5_1 and 15_1 ratios), thus, suggesting a better overlap of the Fmoc groups. On the other hand, the absorption bands in the 250–295 nm region are generated by the transfer of chirality to fluorenyl fragments [31]. The four compounds exhibit similar dichroic behavior. It is observed that as the amount of lysine increases, the signals become positive due to the intramolecular interactions that take place. This reversal of the CD signal can be attributed to an apparent reversal of the amino acid configuration caused by their orientation. The Lys_Ser, Lys_3Gly and Lys_Glu samples co-assembled in a gravimetric ratio of 2_1 show the presence of a positive peak at 254 nm, followed by a negative peak at 274 nm with strong absorption in the case of the Lys_Glu sample (in agreement with rheological data which attest a strong network in the case of co-assembly with Fmoc-Glu). According to literature data [32], the presence of negative peaks in the CD spectrum indicates the ordering and formation of 3D structures. Co-assembly of 5_1 and 15_1 amino acid gravimetric ratios with only positive peaks in the CD spectrum could suggest a high supramolecular organization of Fmoc aromatic groups by the formation of π-π intermolecular interactions [33].

Given that the intermolecular aromatic interactions of fluorenyl fragments in Fmoc functional molecules play an important role in hydrogel formation, fluorescence spectroscopy was used to understand the molecular arrangement of the two compounds in hydrogels by probing the maximum emission wavelength (max) of the fluorenyl fragment. The fluorescence peak positions of the fluorenyl moiety can be utilized to highlight the aggregation dynamics and the associated nanostructural mechanism underlying the Fmoc modified self-assemblies.

The fluorescence spectra of the gels obtained after 24 h (Figure 4b) show a dominant peak at 325 nm, but with different intensities of fluorescence. The position of the peak in the fluorescence spectrum can indicate the association dynamics and the assembly mechanism of the fibrillar nanostructures underlying the structuring process. The co-assembled Lys_Glu has maximum intensity, while the mixture with 3Gly peptide in the solution has low intensity. The spectral pattern did not change with time, indicating that the self-assemblies were considerably stable.

The absorption spectra of the solutions of the co-assembled structures are presented in Figure 4c. There is a significant absorption between 250 and 300 nm due to emission from fluorenyl excimer species. It is also observed that the presence of charged amino acids (such as Glu) in the pair causes an increase in absorbance. When using amino acids with nonpolar radicals (Gly) or neutral polar radicals (Ser), the absorbance is lower [34].

The UV-VIS absorption spectra of the Lys_Glu and Lys_3Gly supramolecular co-assembly in a 5_1 gravimetric ratio show a λ max at 260 nm, whereas, in the case of the co-assembled Lys_Ser hydrogel, an additional weak peak appears at 300 nm. This pattern can also be seen in the spectrum of the self-assembled hydrogel Lys (in detail), which suggests an antiparallel molecular arrangement of Fmoc aromatic fragments.

#### 3.2.5. SEM Analysis

**Figure 5:**
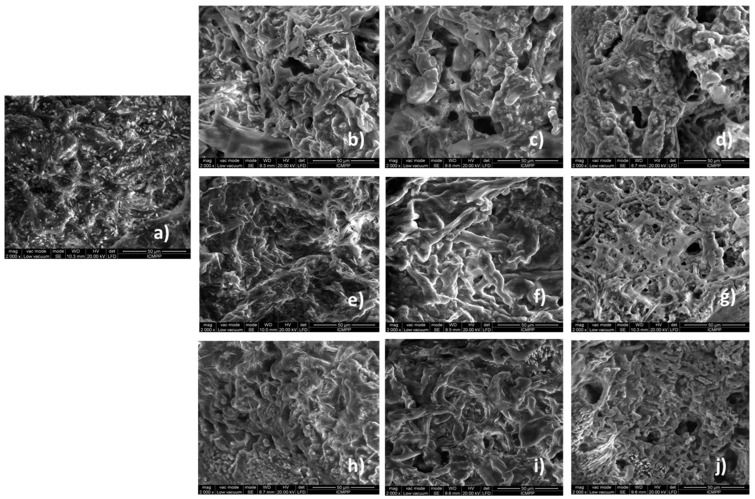
SEM microscopy of (**a**) Lys, (**b**) Lys_Ser_15_1, (**c**) Lys_Ser_5_1, (**d**) Lys_Ser_2_1, (**e**) Lys_Glu_15_1, (**f**) Lys_Glu_5_1, (**g**) Lys_Glu_2_1, (**h**) Lys_3Gly_15_1, (**i**) Lys_3Gly_5_1 and (**j**) Lys_3Gly_2_1.

To gain further insight into the effect of the co-assembly process on the network structure, SEM was used to investigate the structures on a nano- and microscale [35]. The synthesized gels are the result of the formation of fibrous network structures, as can be seen from SEM microscopy of the corresponding freeze-dried gels (Figure 5). The Lys-_Ser shows aligned fibers after drying, which corresponds to a tubular micellar system, as opposed to a cross-linked fibrillar network, as in the case of gels with Lys_Glu and Lys_3Gly.

If we compare the co-assembled structures with the blank (only Lys gel), it is observed that the gels obtained by co-assembly have a skin-like fibrous morphology with two types of fibers that differ in the recorded diameters. The fact that the diameter of the fibers is larger compared to the supramolecular structures based on Fmoc-Lys-Fmoc suggests the formation of a relatively homogeneous network, generated by the interactions between the two components.

The performance of the gels is based on structural characteristics, such as chemical nature, porosity and dimensions of pores [36]. The pores of the freeze-dried gels have specific shapes and dimensions, in direct relation to their chemical composition. In the case of gels obtained by co-assembly, the formation of the network requires additional attention. The sample with Glu and 3Gly shows a more compacted fibrillar network compared to the other samples. In the case of the Lys_Ser gels, homo-aggregates or hetero-aggregates appear, especially at the ratio of 5_1. However, all samples present at a ratio of 5_1 between components a more irregular aspect with higher fibrils. Therefore, SEM results suggest that the overall morphology of Lys_X hydrogels is fibrous in nature, while the intrinsic morphology of these gels is different, which could be attributed to the difference in the molecular arrangements of the assemblies.

### 3.3. Rheological Studies

**Figure 6:**
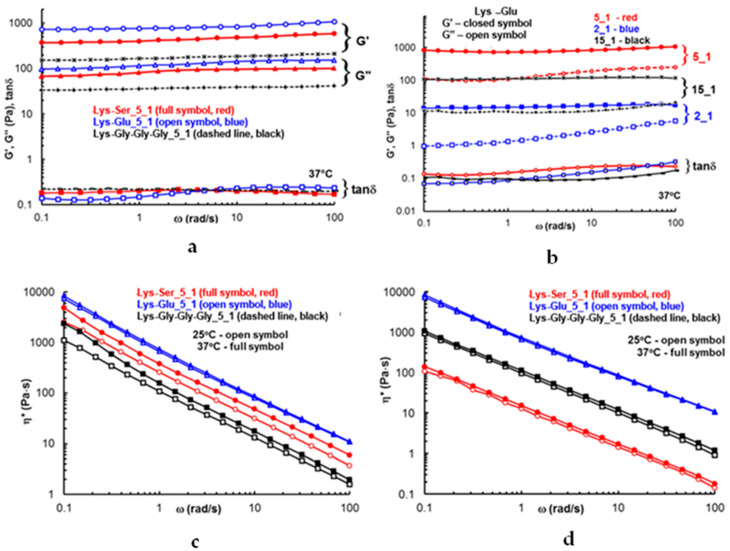
The viscoelastic parameters (G’, G” and tanδ) (**a**,**b**) and complex viscosity (**c**,**d**) as a function of oscillation frequency for Lys-based samples for a constant ratio of 5_1 between the components (**a**,**c**) and for Lys_Glu pairs at different ratios (**b**,**d**), at 37 °C and 25 °C, as shown in each plot.

The formation of the network structure was also confirmed by rheological investigations. Figure 6a,b shows the viscoelastic moduli (G’ and G″) and the complex viscosity determined as a function of the oscillation frequency. All investigated samples showed a gel-like behavior (G’ > G″, with G’ and G″ approx. independent of ω, and tan δ < 1). The modulus of elasticity, as a measure of the gel strength, is influenced by the composition. The Lys_Glu sample presents the highest elastic modulus, which confirms a better structuring and strength of this gel [37]. Regarding the influence of the ratio between the components, there is a stronger structuring in the case of the Lys_Glu sample, with a ratio of 5_1. However, a gel-like behavior (G’ > G″, and tan δ approximately 0.1–0.2) can be observed for all analyzed samples (Table 1), which proves the occurrence of the co-assembly process and the quality of LMWGs in the studied systems. A higher viscosity was registered in the case of Lys_Glu (Figure 6c,d), which highlights the positive role of Fmoc-Glu in structuring the network. Furthermore, temperature plays a more significant role only in the cases of Lys_Ser and Lys_3Gly (Figure 6c), when the co-assembly process was improved by increasing the temperature from 25 °C to 37 °C.

In the case of Lys_Glu, we detected negligible changes in the temperature (Figure 6d).

## 4. Conclusions

Briefly, new systems of LMWGs obtained by co-assembling Fmoc-Lys-Fmoc with other amino acids (Ser, Glu) or short peptides (3Gly) have been designed. These structural units can promote supramolecular co-assembly in both aprotic and protic solvents by π-π stacking, multiple hydrogen bonding, and van der Waals interactions. Co-assembly capacity was assessed by combining different characterization techniques: DLS, FTIR, SEM microscopy, fluorescence dichotomy, rheology and thermal analysis. DLS studies confirm that the supramolecular structure occurs in time. FTIR spectroscopy and SEM microscopy data support the formation of inter- and intramolecular physical bonds that ensure the formation of fibrils and their organization in the 3D network. This observation is also confirmed by rheological studies, which show the gel-like behavior (G’ > G″ and tan δ approximately 0.1–0.2) being observed for all samples, which proves the occurrence of the co-assembly process and the quality of LMWGs in the studied systems. The Lys_Glu presents the best rheological characteristics, which is in accordance with dichroism, fluorescence and SEM observations.

## Figures and Tables

**Table 1:** The composition of the samples investigated in this study.

Sample	Sample Code	Composition for 5 mL of Gel			
		Fmoc-Lys-Fmoc g	Second Compound g	DMSO µL	Water mL
Fmoc-Lys-Fmoc	Lys	0.0250	-	120	4.88
Fmoc-Lys-Fmoc _Fmoc-Ser_2_1	Lys_Ser_2_1	0.00625	0.00375	60	4.94
Fmoc-Lys-Fmoc _Fmoc-Ser_5_1	Lys_Ser_5_1	0.0125	0.0025	80	4.92
Fmoc-Lys-Fmoc _Fmoc-Ser_15_1	Lys_Ser_15_1	0.0187	0.00125	110	4.89
Fmoc-Lys-Fmoc _Fmoc-Glu_2_1	Lys_Glu_2_1	0.00625	0.00375	60	4.94
Fmoc-Lys-Fmoc _Fmoc-Glu_5_1	Lys_Glu_5_1	0.0125	0.0025	80	4.92
Fmoc-Lys-Fmoc _Fmoc-Glu_15_1	Lys_Glu_15_1	0.0187	0.00125	110	4.89
Fmoc-Lys-Fmoc_Fmoc-Gly-Gly-Gly_2_1	Lys_3Gly_2_1	0.00625	0.00375	60	4.94
Fmoc-Lys-Fmoc_Fmoc-Gly-Gly-Gly_5_1	Lys_3Gly_5_1	0.0125	0.0025	80	4.92
Fmoc-Lys-Fmoc_Fmoc-Gly-Gly-Gly_15_1	Lys_3Gly_15_1	0.0187	0.00125	110	4.89

**Table 2:** Name and structure of compounds used in the co-assembled process.

Name and Structure of First Compound	Name and Structure of Second Compound	Images of Co-Assembled Gels	Conclusions
Fmoc-Lys-Fmoc 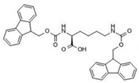	Fmoc-Gly-Gly-Gly 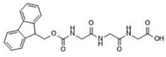	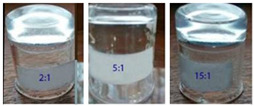	Stable gels
Fmoc-Lys-Fmoc 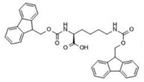	Fmoc-Glutamic acid 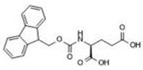	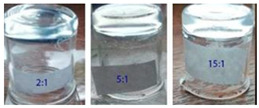	Stable gels with translucent aspect
Fmoc-Lys-Fmoc 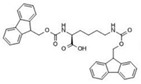	Fmoc-serine 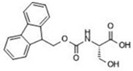	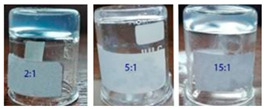	Stable gels with opaque aspect

**Table 3:** The main parameters of the thermal analysis.

Sample	Degradation Stage	T_onset_ (°C)	T_peak_ (°C)	T_endset_ (°C)	W (%)	Residue	T_10_ (°C)	T_20_ (°C)
*Lys*	I II III IV V	62 182 262 316 361	144 189 280 322 365	172 196 307 350 398	10.44 33.00 8.77 6.85 6.76	34.18	178	193
*Lys_Ser*	I II III IV	27 187 213 305	138 197 280 323	183 204 303 357	9.82 15.10 21.03 11.28	42.77	190	206
*Lys_3Gly*	I II III IV	62 89 188 283	74 128 201 307	80 172 220 335	1.71 6.04 26.18 11.49	54.58	195	209
*Lys_Glu*	I II III IV	27 115 194 270	97 132 211 311	109 174 240 334	3.98 5.73 34.55 17.57	38.17	183	202

## Data Availability

The authors confirm that the data supporting the findings of this study are available within the article.
